# Tissue Distribution and Efficacy of Gold Nanorods Coupled with Laser Induced Photoplasmonic Therapy in Ehrlich Carcinoma Solid Tumor Model

**DOI:** 10.1371/journal.pone.0076207

**Published:** 2013-10-02

**Authors:** Mostafa A. El-Sayed, Ali A. Shabaka, Osama A. El-Shabrawy, Nemat A. Yassin, Sawsan S. Mahmoud, Siham M. El-Shenawy, Emad Al-Ashqar, Wael H. Eisa, Niveen M. Farag, Marwa A. El-Shaer, Nabila Salah, Ahmed M. Al-Abd

**Affiliations:** 1 Laser Dynamics Laboratory, School of Chemistry and Biochemistry, Georgia Institute of Technology, Atlanta, Georgia, United States of America; 2 Department of Laser Physics, National Research Center, Giza, Egypt; 3 Department of Pharmacology, National Research Center, Giza, Egypt; 4 Department of Pathology, National Research Center, Giza, Egypt; 5 Department of Pharmacology, Faculty of Pharmacy, King Abdulaziz University, Jeddah, Kingdom of Saudi Arabia; Argonne National Laboratory, United States of America

## Abstract

Gold nanorods (GNR) within tumor microregions are characterized by their ability to absorb near IR light and emit heat in what is called photoplasmonic effect. Yet, the efficacy of nanoparticles is limited due to intratumoral tissue distribution reasons. In addition, distribution of GNRs to normal tissue might result in non specific toxicity. In the current study, we are assessing the intratumoral and tissue distribution of PEGylated GNRs on the top of its antitumor characteristics when given intravenously or intratumoral to solid tumor bearing mice and coupled with laser photoplasmonic sessions. PEGylated GNRs with a longitudinal size of less than 100 nm were prepared with aspect ratio of 4.6 showing strong surface plasmon absorption at wavelength 800 nm. Pharmacokinetics of GNR after single I.V. administration (0.1 mg/kg) showed very short systemic circulating time (less than 3 h). On the other hand, tissue distribution of I.V. GNR (0.1 mg/kg) to normal animals showed preferential deposition in spleen tissue. Repeated administration of I.V. GNR resulted in preferential accumulation in both liver and spleen tissues. In addition, I.V. administration of GNR to Ehrlich carcinoma tumor bearing mice resulted in similar tissue distribution; tumor accumulation and anti-tumor effect compared to intratumoral administration. In conclusion, the concentration of GNR achieved within tumors microregions after I.V. administration was comparable to I.T. administration and sufficient to elicit tumoral growth arrest when coupled with laser-aided photoplasmonic treatment.

## Introduction

Cancer is so far a national and international health problem [1]. Over more than 5 decades mortality due to cancer, nonetheless solid tumor remains constant regardless of the discovery of several dozens of novel anticancer drugs (natural and synthetic). Due to the unique properties of solid tumor microenvironment, the poor intratumoral drug distribution represents major factor in drug availability at the target site [2]. Resistance of solid tumor to anticancer treatment has been attributed currently to pharmacokinetic reasons rather than resistance at the cellular level [3,4]. Anticancer agent needs to be distributed preferably to solid tumor regions to generate tumor killing effect [3,5,6]. Recent discoveries for the treatment of solid tumor were highly dependent on new and sophisticated molecules such, as siRNA [7], aptamers [8], monoclonal antibodies [9] and nanoparticles [10]. Studying the pharmacokinetics and tumor/tissue distribution of these modern anticancer modalities are inevitable for full evaluation of their potential anticancer effect [11].

Nanomaterials have diverse effects that draw the attention of scientists from different specialties. Attributed to their unique properties, nanomaterials have been involved in numerous applications including biomedical uses [12]. Inorganic nanoparticles have been increasingly obtaining the attention as potential diagnostic and therapeutic tools in the field of oncology. Nonetheless, the use of fluorescent quantum dots, carbon nanotubes, gold nanoparticles, iron oxide magnetic nanobeads and ceramic nanoparticles is growing in the fields of tumor targeting, imaging, photothermal therapy and drug delivery systems [13].

Gold nanoparticles are subclass of nanomaterials intensively investigated for biomedical uses which is attributed to their excellent biocompatibility with human tissues. Moreover, gold nanoparticles is currently approved by the United States Food and Drug Administration for the treatment of rheumatoid arthritis [14]. Importantly, gold nanorods have surface plasmon resonance band at the near infra-red region, in which light photons penetrate deeply into biomatrices and got converted into heat in a so-called 'photothermal effect' [15]. In addition, Gold nanorods (GNRs) can be used dually for tumor imaging and treatment [16,17]. GNR, attributing to this characteristic phenomenon, shows great potential in the fields of photothermal therapy for the treatment of cancer [18]. The photodynamic therapy (PDT) employs light absorbing photosensitizing dyes in order to generate reactive oxygen species *in-situ* for achieving damage of superficial tumors. Herein, recent advances in the field of nanoscience employs noble metal nanostructures such as gold nanorods with unique photoplasmonic properties, well suited for applications in cancer photothermal therapy [19]. Plasmonic photothermal therapy with gold nanostructures has been used previously to generate significant heat within tumors for tissue ablation and anticancer effect [20,21].

Herein, we have evaluated the tumor and normal tissue distribution of gold nanorods injected systemically in normal and tumor bearing animals. In addition, we assessed the antitumor effect of systemically administered gold nanorods coupled with near IR laser plasmon photothermal therapy in subcutaneously transplanted Ehrlich carcinoma solid tumor model. 

## Results

### Preparation and characterization of GNRs

**Figure 1:**
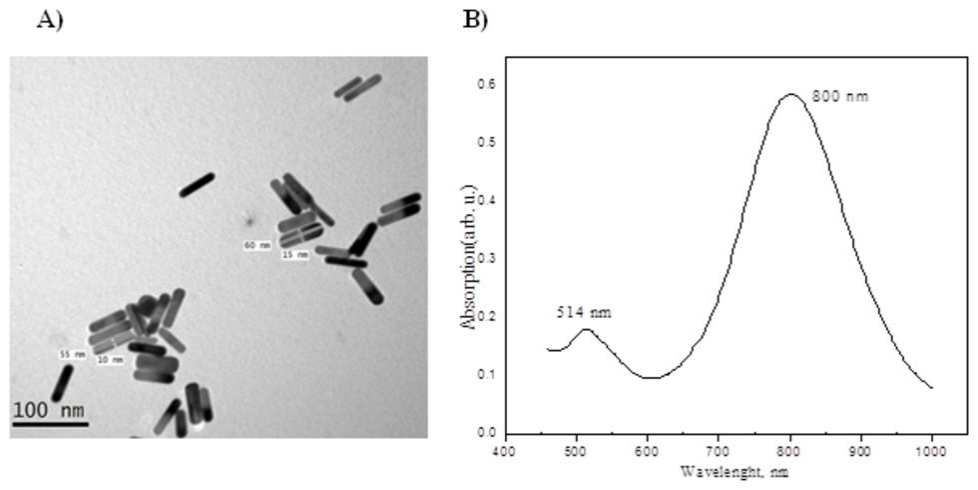
Physical properties of GNRs. TEM image of GNRs with Plasmon band energies at 800 nm (A) and UV- Visible NIR absorption spectra of the GNRs (B) prepared using single surfactant mixtures. Scale bar = 100 nm.

Transmission electron microscope (TEM) image of the prepared GNRs were shown in Figure (1-A). From this image, it is clear that, a uniform shape of GNRs with regular distribution were formed. The aspect ratio is defined as the length of the major axis divided by the width of the minor axis. The length of rods (60 ± 5 nm) and aspect ratio is (4.6). The 4.6 aspect ratio rods were too far in size from the spheres to allow for efficient separation. The formation mechanism of GNRs depend on the template, we have observed that using concentrated CTAB solution enhances the rod yield. Concentrated CTAB has a tendency to form elongated rod-like micelle structures that possibly assist in rod formation, as well as stabilizing the rods. This template was used earlier for the electrochemical synthesis of GNR, and the aspect ratio was controlled by introducing Ag^+^ ions or a more hydrophobic cosurfactant (compared to CTAB). The enhanced growth rate in the presence of seed (possibly diffusional growth) and the rod-like micellar template contribute to the rod formation.

The optical properties of metallic nanoparticles depend on shape. This is due to the absorption of visible light both along the length of the nanorod (the longitudinal plasmon band) and along the width of the nanorod (the transverse plasmon band). The ultraviolet visible (UV–VIS) spectra of the GNRs in deionized water as a solvent were shown in Figure (1-B). In this figure, it was found that, the surface plasmon absorption of gold nanorods have two bands. A strong long-wavelength band (800 nm) attributed to the longitudinal oscillation of electrons and a weak short-wavelength band (514 nm) due to the transverse electronic oscillation was observed.

### Tissue distribution of GNRs after single I.V. administration

**Figure 2:**
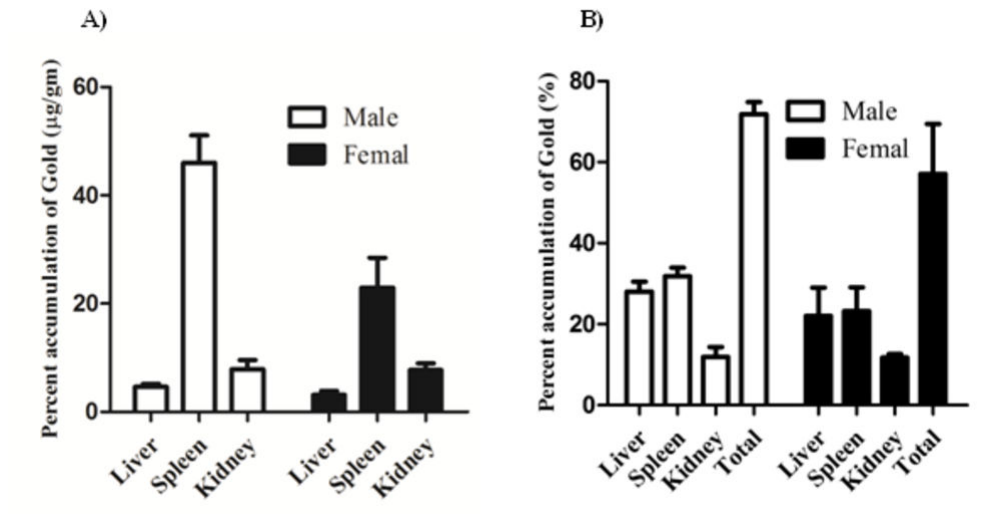
Tissue distribution of GNRs after single I.V. administration to male and female normal animals. GNRs were administered by I.V. injection (0.1 mg/kg) and assayed after two weeks in major excretory organs (liver, spleen and kidney). Concentration of GNRs (A) and the percent residual amount of the total administered dose (B) are presented. Data are presented as mean ± SEM (n=6).

After single I.V. administration of GNRs to normal male and female animals, preliminary distribution into different major organs (liver, spleen and kidney) was evaluated after two weeks of exposure (Figure 2). The maximum concentration of GNRs was achieved in spleen tissue of male and female animals; however significantly higher in male than female animals (2 fold). Concentration of GNRs was comparable between male and female animals in liver and kidney tissues. In male and female animals, concentrations of GNRs in spleen tissue were 9.9 and 7.3 folds higher than in liver tissue of male and female animals, respectively (Figure 2-A). However, the percent of GNRs accumulation in liver and spleen tissues of male (28.1±2.4% and 31.8±2.1%, respectively) and female (22.1±6.9% and 23.2±5.9%, respectively) animals were not significantly different. That might be attributed to the greater relative organ mass between liver and spleen. It is worth mentioning that, 71.3±3.01% and 57.1±12.3% of total administered dose of gold NRs were retained within the liver, spleen and kidney tissues after two weeks of single I.V. administration in male and female animals, respectively (Figure 2-B).

### Tissue accumulation of GNRs after repeated administration

**Figure 3:**
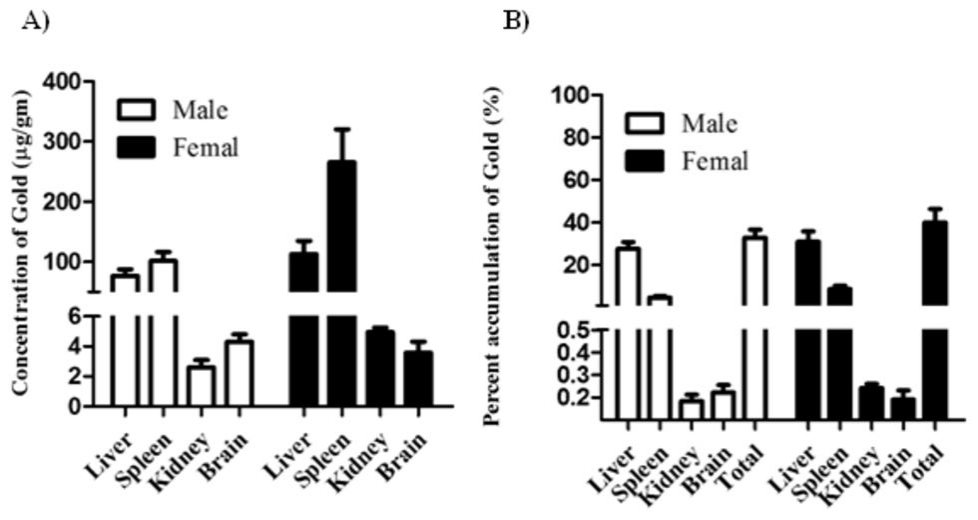
Tissue accumulation of GNRs after repeated I.V. administration to male and female normal animals. GNRs were administered by I.V. injection (0.1 mg/kg for five consecutive days of each month and repeated for 6 months). Three week after the last injection major target organs (liver, spleen, kidney and brain) were assayed for tissue concentration of GNRs (A) and the percent residual amount of the total administered dose (B). Data are presented as mean ± SEM (n=6).

In clinical setting, chemotherapy and radiotherapy are usually prescribed in the form of treatment cycles to achieve maximum tumor killing effect. In this context, the accumulation of GNRs has been studied after repeated I.V. administration (0.1 mg/kg) into normal animals with special emphasis on liver, spleen, kidney and brain tissues (Figure 3). Similarly, the maximum concentration of GNRs was achieved in spleen tissue of male and female animals. In contrast to acute exposure, concentration of GNRs was significantly higher in female than male animals (2.6 fold). Concentration of GNRs was comparable between male and female animals in liver, kidney and brain tissues. Interestingly, concentration of GNRs was comparable in kidney and brain tissues of male (2.6±0.5 and 4.3±0.5 µg/g, respectively) and female (4.9±0.3 and 3.6±0.7 µg/g, respectively) animals and substantially lower than its concentration in liver and spleen tissues in male (76.7±11.1 and 101.7±14.8 µg/g, respectively) and female (112.6±22.4 and 265.5±54.9 µg/g, respectively) animals, respectively (Figure 3-A). In contrary to acute exposure, the percent of GNRs accumulated in liver tissues of male and female (27.7±3.2% and 30.0±4.8%, respectively) animals was significantly higher than accumulated in spleen tissues (4.7±0.7% and 8.7±1.6% in males and females, respectively). It is worth mentioning that, total accumulation of GNRs after repeated administration was substantially lower than after single administration in male and female animals, respectively (Figure 3-B).

### Tissue distribution of GNRs in tumor bearing animals

**Figure 4:**
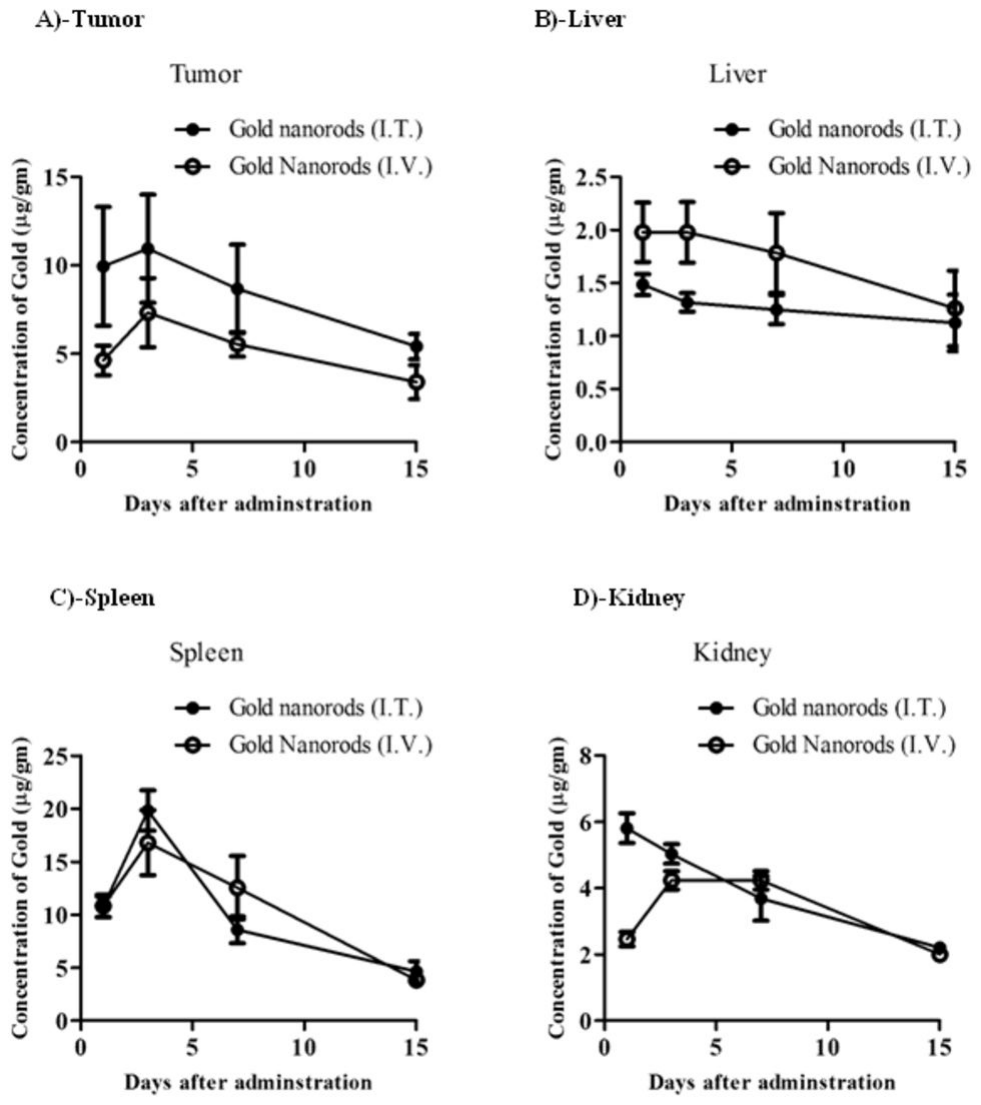
Tissue pharmacokinetics of GNRs after I.V. and I.T. administration to EACC tumor bearing mice. GNRs were administered by I.V. (◌) or I.T. (●) injection (1.5 mg/kg) to tumor bearing mice. Tissue concentration of gold in tumor (A), liver (B), spleen (C), and kidney (D) tissues were assayed for Gold conetnt at different time intervals until two weeks. Data are presented as mean ± SEM (n=3).

To assess tumor/normal tissue distribution of GNRs in tumor bearing animals, a dose of 1.5 mg/kg was administered intratumoral (I.T.) or intravenous (I.V.) and tissue concentration of GNRs was determined in tumor, liver, spleen, and kidney tissues at different time intervals (Figure 4). GNRs injected via I.T. route demonstrated apparently higher concentration within tumor tissue than I.V. route; however, was not significantly different at all time points examined. In both I.T. and I.V. treatments, GNRs concentration in tumor tissue was elevated to achieve C_max_ after 72 h of injection (Figure 4-A). Later on, after 72 h, elimination phase from tumor tissue started with calculated T_1/2_ of 13.8 and 11.6 days for I.T. and I.V. routes, respectively. Despite the comparable tumor concentrations of gold NRs between I.T. and I.V. administration, AUC was higher (1.6 folds) after I.T. than I.V. administration (Table 1). On contrary, GNRs injected via I.T. route demonstrated apparently lower concentration than I.V. route within the rest of normal tissues (liver, spleen and kidney) under investigation; however, was not significantly different at all time points examined. Similar to tumor tissue, after I.T. and I.V. treatments with GNRs, the concentration in almost all normal tissues was elevated to achieve C_max_ after 72 h of injection (Figure 4-b-D). Later on, after 72 h, elimination phase from tumor tissue started with calculated T_1/2_ in different organs ranging from 11.6-69.3 days (Table 1). It is worth mentioning that elimination phase in kidney started in animals treated with I.T. GNRs after 24 h (Figure 4-D). Interestingly, tissue exposure of GNRs in normal tissues under investigation was comparable (comparable AUC) after I.T. and I.V. administration (Table 1).

**Table 1:** Tissue distribution of gold nanorods after I.V. and I.T. administration in tumor bearing animals.

**Tissue**	C_max_		AUC_0-last_		AUC_0-inf_		Elimination half life time (days)		C_max_ ratio	Tissue exposure ratio (TER)^*^	Tissue Exposure Index (TEI)^†^	
	(µg/g tissue)		(day. µg/g tissue)		(day. µg/g tissue)							
	I.V.	I.T.	I.V.	I.T.	I.V.	I.T.	I.V.	I.T.			I.V.	I.T.
**Tumor**	7.3	15.14	75.7	121.4	101.2	162	11.6	13.8	2.1	1.6	100	100
**Liver**	2.0	1.5	24.6	18.2	34.1	26.6	23.1	69.3	0.75	0.74	32.5	15.0
**Spleen**	16.8	19.8	157.1	145.6	185.8	180.4	4.1	6.3	1.2	0.93	207.5	119.9
**Kidney**	4.2	5.8	49.8	54.8	64.8	71.3	11.6	11.6	1.4	1.1	65.8	45.1

* Tissue exposure ratio is calculated as ratio between AUC_I.T_ /AUC_I.V_ and indicates the relative degree of exposure of each tissue to gold nanorods when administered locally or systemically.

† Tissue Exposure Index is calculated as ratio between AUC of gold nanorods in normal tissue relative to tumor tissue and indicates the relative degree of tumor tissue targeting.

Tissue exposure index (TEI) represents the relative normal organ exposure to GNRs to tumor tissue after I.T. or I.V. administration. Liver and kidney tissues showed TEI indicative of lower organ/tumor exposure characteristics after either I.T. or I.V. administration (TEI ranged from 14.99-65.79%). TEI of spleen indicated higher organ/tumor exposure and was higher in I.V. than I.T. administration showing 207.5 and 119.9%, respectively (Table 1).

Relative tissue exposure ratio (TER) is a parameter to present the relative overall organ exposure to GNR after I.T. administration relative to I.V. administration (Table 1). It was calculated as the ratio of AUC_0-last_ after I.T. administration to the AUC_0-last_ after I.V. administration for all studied tissues. GNRs exposure in tumor was 1.6 folds higher after I.T. compared to I.V. adminstration, however, not more than 1.1 folds higher in any other organ indicating that I.T. administration *per-se* might improve drug distribution to tumor tissue. TER in all other tissues under investigation ranged from 0.7 to 1.1 (Table 1), indicating that exposure in these well perfused normal tissues to GNRs can be minimally attributed to the route of injection (Table 1). It is worth mentioning that, transient tumor exposure (C_max_ ratio) to GNRs after I.T. was 2 folds higher that I.V. administration while ranged from 0.8-1.4 for other normal tissues under investigation (Table 1). These data suggests that administration route (I.T. or I.V.) of GNRs might not significantly influence their anti-tumor efficacy due to pharmacokinetics reasons.

### Anti-tumor activity of GNRs coupled with laser plasmonic therapy

**Figure 5:**
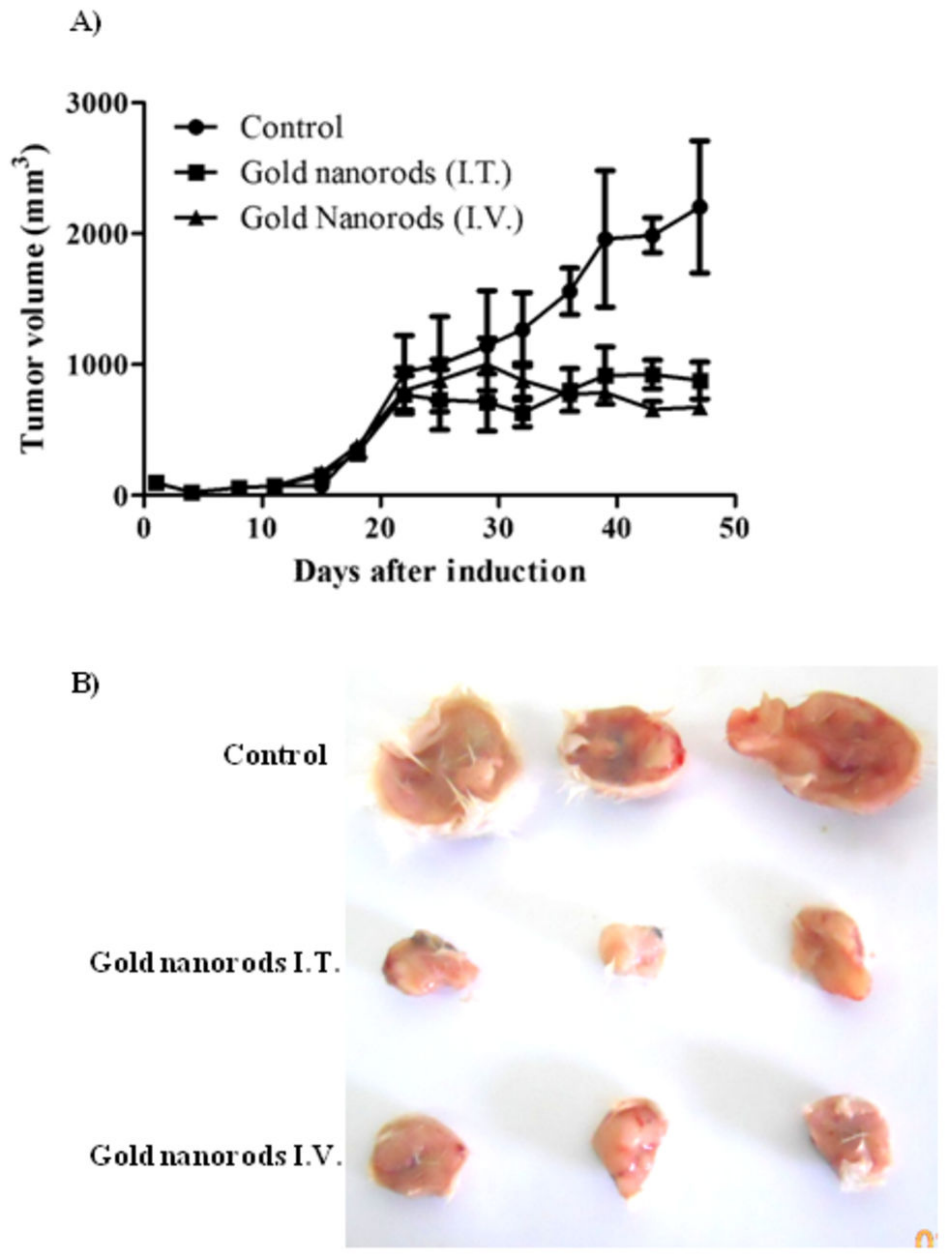
Antitumor activity of GNRs coupled with laser induced photo plasmonic thermal therapy in EACC solid tumor bearing mice. EACC tumor bearing mice were given gold NRs (1.5 mg/kg every three weeks) by I.V. (▲) and I.T. (■) administration compared to PBS treated animals (●). Animals were exposed to laser plasmonic beam (50 W/cm^2^ for 2 min) every week. Tumor size was measured every three days and plotted (A). Representative tumors are shown in panel (B). Data are presented as mean ± SEM (n=10).

To assess the anti-tumor effect of GNRs coupled with PPT, 1.5 mg/kg GNRs was injected I.T. and I.V. every three weeks to EAC tumor bearing mice, exposed to near IR laser beam (50 W/cm^2^ for 2 min) and tumor growth rate was measured for up to 47 days. Immediately after the session, tumor tissue was overheated to about 79 °C in the center of laser beam exposure. The temperature of the tumor tissue declined outward to reach about 41 °C at tumor boundaries (Figure S1). GNRs coupled with PPT substantially arrested EAC tumor growth from day 22 to day 47 without any significant change in tumor size. Eventually, untreated mice showed continuous tumor growth to reach at day 47 about 6.3 folds the initial tumor size at the start day of treatment (day 18). Tumor size throughout the whole duration of treatment (day 22 – day 47) did not show any significant difference between I.T. and I.V. treatment groups (Figure 5).

### Pathological features of solid tumor after treatment with GNRs coupled with laser plasmonic therapy

**Figure 6:**
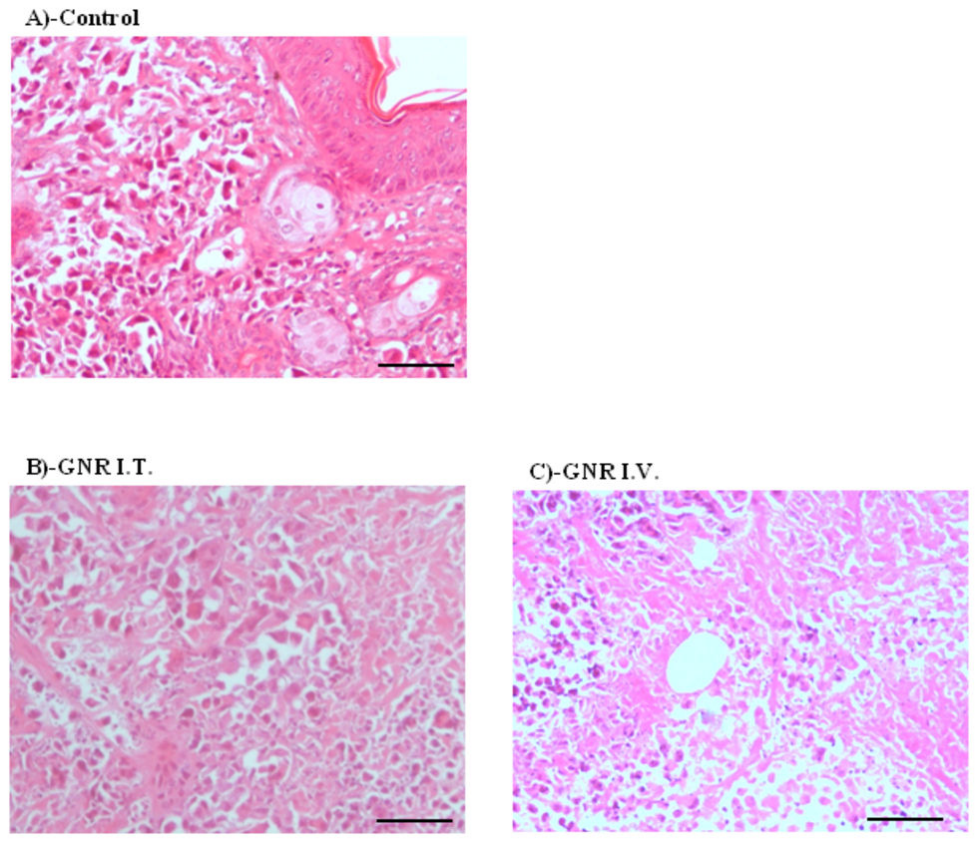
Histological examination for EACC solid tumor treated with gold NRs coupled with laser induced photo plasmonic thermal therapy. EACC tumors of control group (A); gold NRs IT treated group (B); and IV treated group (C) were stained by H&E regular stain. GNR coupled with PTT showed massive tissue destruction appeared as non-cellular debris eosinophilic areas (arrows) Scale bar = 20 µm.

To assess the pathological changes in solid tumor after treatment with GNRs coupled with laser plasmonic therapy, the cellular, vascular and stromal compartments were examined in H&E stained tumor sections. Solid Erlich carcinoma in the control group appeared as compact subcutaneous masses of tumor cells which invade the subjacent connective tissue. Tumor cells were highly cellular anaplastic, atypical pleomorphic, polygonal, with abundant eosinophilic cytoplasm and prominent central nuclei. The surrounding stroma was reduced with some inflammatory cell infiltration (Figure 6-A).

In tumors treated with direct I.T. GNR coupled with laser plasmonic therapy, remarkable cellular debris (indicated by arrows) was observed in addition to profound desmoplastic stroma in the form of fibrocollagenous bundles and peri-vascular hyalinosis. These findings might explain the regression of tumor growth and further tumor invasion to the surrounding connective tissues. In addition, the marked lymphocytic infiltration was noticed in the surrounding stroma (Figure 6-B).

Intravenous GNRs coupled with laser plasmonic therapy showed abundant tumoral cell debris, more oesinophilic tumor cells (indicated by arrows) and peri-vascular hyalinosis indicative of nuclear damage. Marker stromal desmoplastic fibrotic bundles were observed which might explain the limitation of tumor invasion (Figure 6-C).

## Discussion

Multifunctional nanoparticles, nonetheless, gold nanorods are designed to take over various functions in the field of oncology, such as tumor targeting, imaging and selective therapy, which offer great promise for the future of cancer prevention, diagnosis, imaging and treatment [16]. Gold nanorods have strong absorbance in the near infrared region, which penetrates deeply into tissues, where the absorbed light energy is converted into heat [22]. These unique photoplasmonic properties comprise promise for photothermal therapy of solid tumors. In the current work we designed, synthesized and purified homogenous GNRs (60 ± 5 nm) with aspect ratio of 4.6 coated with PEG molecules for enhanced biocompatibility. The surface plasmon absorption of the prepared GNRs has two bands at 800 nm and 514 nm which were attributed to the longitudinal and the transverse electronic oscillation, respectively. Apart from the shape, size and aspect ratio of GNRs which influence their photoplasmonic properties [23]; the degree of PEGylation and chain length of PEG affect the biocompatibility of GNRs like other nanomaterials [24]. Accordingly, the current formulation of GNRs comprises unique and tunable nanostructure with great potentiality in photoplasmonic therapy of solid tumors.

Apart from photonic physical properties, biocompatibility and potential toxicity of GNRs constitutes obstacle in front of using nanometalic structures as a therapeutic remedy. Not only the metal itself, but also chemical materials used in the synthesis such as CTAB, might carry some toxic effect *in-vivo* [25]. In the current work, no serious signs of toxicity were observed in normal animals treated with very high doses (13.5 mg/kg) of GNRs in terms of vital organs such as, liver and kidney functions (unpublished observation). Much higher doses of gold nanoparticles (2700 mg/kg) appeared non toxic to experimental animals as well [26]. Yet, *in-vivo* toxicological studies indicated no mortalities, significant weight changes nor adverse effects in mice treated with PEGylated GNRs [27]. Toxicity of GNRs and other nanomaterials directly correlates to accumulation in vital organs, nonetheless, reticuloendothelial system such as, liver and spleen [28]. In addition, GNRs (single and repeated administration) substantially accumulated in liver and spleen of normal animals. No significant difference in accumulation kinetics of GNRs between male and female normal animals after single or repeated administration. The level of GNRs in brain tissues of male and female animals after repeated administration was relatively lower than other tissues. Yet, accumulation of GNRs in these tissues has not been reflected in the form of any behavioral or neurological sign or symptoms. However further detailed behavioral neurological studies are strongly recommended. Another interesting observation; constant concentrations of GNRs were observed in kidney tissue after single and repeated administration regardless of the significantly different dose burden (30 folds) between both situations. This could be attributed to the renal elimination of GNRs as described in previous reports [26]. The higher tissue concentration/accumulation of GNRs in liver and spleen tissue might reflect the higher saturation concentration of these reticuloendothelial systems to nanomaterials such as GNRs [28].

In tumor bearing animals, GNRs accumulated in tumor tissue comparably after I.V. and I.T. administration, as well as, in the rest of all examined tissues (liver, spleen, kidney and brain). Surprisingly in the current work, PEGylated GNRs disappeared from plasma only after 3 h of I.V. administration (data not shown). In general, prolonging the circulating time of GNR’s via PEGylation was considered as a tool to achieve better tumor tissue accumulation [29]. However, the current formulation of GNRs attained exelent tumor tissue accumulation kinetics. Other GNRs (6.1 aspect ratio) was reported to be cleared from the blood after 3 days, however still detected in different tissues [22]. According to our previous observation and others, comparable accumulation of GNRs in tumor tissues after I.T. and I.V. might be attributed to enhanced permeation and retention effect; the perforated leaky tumoral blood vessel allow macromolecules to accumulate passively in the tumor micro-milieu [22,30]. Passive targeting of GNRs and other nanomaterials would be tunable by the aid of modifying shape, size, aspect ratio and degree of PEGylation [31]. On the other hand, active targeting of GNRs using antibodies, aptamers, or other sophisticated bioligands comprises important tool for tumor targeting [16,21,32,33]. In the current work, close TER of GNRs in tumor tissue indicates that active targeting for the current GNRs formulation in not crucial for tumor targeting. Several other formulations of GNRs (different size and aspect ratio) could not achieve satisfactory intra-tumural concentration and anti-tumor effect by passive targeting after I.V. administration [29]. Active tumor targeting in several circumstances does not comprise significant advantage *in-vivo* as well [28,34]. Interestingly, it was reported that passive cellular targeting might be influenced solely by tuning the shape and size of gold nanoparticles [31,35]. Elimination rate of GNRs from all organs was similar after I.V. and I.T. administration (except liver) with maximum of 138-161 days for complete clearance of GNRs from all tested organs (6-7 elimination half life times are required for complete clearance from specific tissue or organ). Longer elimination of GNRs from liver after I.T. administration could be attributed to the depot of GNRs in tumor tissue after I.T. administration and redistribution process. Similarly, shape and size of GNRs strongly influence their biodistribution to normal tissues. It has been reported that nanorods accumulate to a lesser extent than nanosphers in reticuloendothelial organs such as, liver and spleen [35].

In our previous work, systemically administered GNRs coupled with near IR laser plasmon therapy efficiently diminished the growth of squamous cell carcinoma [36]. Herein, we are expanding our work to Ehrlich carcinoma solid tumor model in pharmacokinetics guided experimental design. Distribution kinetics and elimination of GNRs from tumor tissue lead us to design the interval of dose repetition (every 3 weeks) as well as the time schedules of laser plasmon exposure (every week). The use of GNRs coupled with near IR laser plasmonic therapy successfully aborted subcutaneously transplanted Ehrlich solid tumor growth kinetics. Dose adjustment of GNRs used in the current study was based on preliminary toxicological study by us (unpublished observation) and by other research groups [26,27]. In addition, the intensity of laser beam used herein (50 W/cm^2^), was very close to the lower threshold energy required for GNRs plasmonic activation that would not be expected to induce any collateral tissue injury. GNRs require 10-10^6^ W/cm^2^ for plasmonic activation *in-situ* [37]. In agreement with this boundaries of laser energy required for plasmonic activation, Huff and coworkers used laser beam of 30 W/cm^2^ to induce hyperthermic effect in tumor cells coupled with GNRs [38]. Based on intratumoral retention (more than 3 weeks), single I.V. or I.T. administration of GNRs would sustain enough intratumoral concentration for 3 weekly consecutive plasmonic exposure sessions (once/week) in a so called “pharmacokinetic guided laser plasmonic therapy”. This approach constituted solid ground from GNRs dosing and laser exposure frequencies. It is worth mentioning that, the ability of the current treatment protocol with GNRs coupled with near IR plasmonic therapy to arrest solid tumor of such big size (300 mm^3^) would pave strong promise for the current formulation in the treatment of superficial malignancies feasible for light exposure. Not only superficial tumors, but also has been suggested by Gu and colleagues to expose deep tissue neoplasia loaded with GNRs to near IR laser beam using endoscopic aided optic fibers in a so called “endoscopic guided microsurgery” [39].

## Conclusion

In conclusion, GNRs administered systemically was equally distributed to Ehrlich carcinoma solid tumor tissues compared to intra-tumoral administration of GNRs. In addition, systemically administered GNRs was equipotent to local intra-tumoral administered GNRs when coupled with laser plasmon thermal therapy in inducing tumor growth arrest. Finally, accumulation of GNRs in vital organs such as liver and spleen apparently was non-toxic; however, might warrant further toxicological detailed studies. Further formulations of better surface plasmon characteristics and less accumulation kinetics in vital organs might pave promise in the field of laser-induced photo plasmon thermal therapy of solid tumors. It is recommend for the current GNRs formulation to be considered for clinical assessment in solid tumor treatment coupled with laser photoplasmonic therapy.

## Materials and Methods

### Chemicals and reagents

Cetyltrimethylammonium bromide (CTAB) and Sodium borohydride (99%) were purchased from Mercy and LOBA chemic respectively. Silver nitrate was purchased from Sigma-Aldrich, L-ascorbic acid. All the reagents were analytical grade, and used without further purification. Deionized water (18 MΩ) was used in all the experiments.

### Preparation of Gold Nanorods

. The nanorods were synthesized according to the seed-mediated growth method [40]. For preparation of NRs, seed and growth solutions were made as described below.

#### Preparation of Seed Solution

CTAB solution (5 mL, 0.20 M) was mixed with HAuCl_4_ (5 mL, 0.0005 M) under vigorous stirring. Next, 0.6 mL of ice-cold 0.01 M NaBH_4_ was added to the solution. The solution turned brownish yellow immediately after adding NaBH_4_, indicating particle formation. The particles in this solution were used as seeds. Vigorous stirring of the seed solution was continued for 2 min. After the solution was stirred, it was kept at 25°C.

#### Growth of GNRs with plasmon bands less than 850nm

In a clean test tube, 10 mL of gentle mixing growth solution, containing (5 mL, 0.20 M) of CTAB and (5 mL, 0.001M) of HAuCl_4_, was mixed with 0.35 mL of 0.004 M AgNO_3_ solution at 25°C. To this solution, 5 mL of 1M HCl was added, and after gentle mixing of the solution 70µL of 0.0788 M ascorbic acid was added. Ascorbic acid as a mild reducing agent changes the growth solution from dark yellow to colorless. It is worth noting that the growth solutions above are identical except for their silver ion content. The final step was the addition of 12µL of the seed solution to the growth solution at 27-30°C. The color of the solution gradually changed within

10-20 min. For longer NRs, the color change takes place more slowly. The temperature of the growth medium was kept constant at 27-30°C in all the experiments. This pathway produces pure NR solutions with aspect ratios 4.6.

#### PEGylation of GNRs

GNRs colloidal solutions were centrifuged twice (20,000g for 15 min) then re-dispersed in deionized water to get rid of excess CTAB. A final concentration of 10 mM mPEG-SH (MW5000, Sigma-Aldrich) and 1 nM colloidal GNRs were mixed. GNR were sonicated overnight and then centrifuged (20,000g for 15 min) and re-dispersed in deionized water to remove non-specifically bound PEG molecules. The PEGylated GNRs were centrifuged (20,000g for 15 min), sterile by filteration (0.22 µm pore size filter), and re-dispersed in sterile 10 mM phosphate-buffered saline (PBS, Gibco) to the desired optical density at 800 nm. Extinction spectra of the PEGylated nanorod saline suspensions showed no peak shift, broadening, or reduction over a 1-week period prior to injection.

#### Instrumentation

Absorption spectra of the prepared solutions were measured in the range of 1000–200nm using Jasco 570 UV–VIS-NIR spectrophotometer. The morphology of gold NRs was studied by Transmission Electron Microscope (JEOL-JEM 2010) operated at 200 kV accelerating voltage. The preparation of TEM grid, the TEM image was taken after separating the surfactant from the metal particles by centrifugation. Typically 1 mL of the sample was centrifuged for 10 min at a speed of 14000 rpm. The upper part of the colorless solution was removed and the solid portion was redispersed in 1 mL of water. 2 µL of this redispersed particle suspension was placed on a carbon coated copper grid and dried at room temperature.

### Animals

Male and female BalbC mice (weight 20-25 g) and Sprauge Dawly rats (weight 120-150 g) were maintained in the pathogen free area of the National Research Center animal house facility (Dokki, Giza, Egypt). Animals had an access to food and water *ad labium*. Animal experimental protocol was approved by the Ethical and Animal Care Committee at the National Research Center.

### Cell culture and subcutaneous induction of tumors

Murine Ehrlich Ascitis Carcinoma cell line, EACC was obtained from National Cancer Institute (Cairo, Egypt), and maintained in RPMI-1640 media supplemented with 100 µg/ml streptomycin, 100 units/ml penicillin and 10% heat-inactivated fetal bovine serum in a humidified chamber at 37°C supplied with 5% (v/v) CO_2_.

For tumor induction, cells were collected and washed 3 times with serum free media, and 1x10^7^ viable cells were injected s.c. into the flank region of each mice.

### Tissue distribution of single I.V. administration of GNRs

To determine the tissue distribution and potential targeting of NRs to major organ of normal animals, rats were given NRs preparation (0.1 mg/kg) intravenousely in PBS solution. Two weeks later, animals were euthanized by cervical dislocation, and these organs (liver, spleen, and kidney) were harvested within 20 min and stored at -80°C to be assayed.

### Tissue accumulation of gold nanorods after repeated administration

To determine the tissue accumulation of NRs in major organs of normal animals, rats were given NR preparation intravenousely in PBS solution (0.1 mg/kg every day for the first 5 days of each month and repeated for 6 months). Three weeks after the last administration, animals were euthanized by cervical dislocation, and their organs (liver, spleen, kidney and brain) were harvested within 20 min and stored at -80°C to be assayed.

### Pharmacokinetics of gold nanorods in tumor bearing animals

When tumor size reached 300 mm^3^, mice were given NR preparation (1.5 mg/kg) either intratumorally or intravenousely in PBS solution. At predetermined time points, mice were euthanized by cervical dislocation, and their organs (tumor, liver, spleen, kidney, brain) were harvested within 20 min and stored at -80°C to be assayed.

### Analysis of gold content

Tissues samples were incinerated for 48 h at 550°C. Ashes were dissolved in concentrated nitric acid and gold content were measured by atomic absorption. Standard curve of HAuCl_4_ were plotted and measured by atomic absorption.

### Assessment of antitumor activity

To evaluate anti tumor efficacy; when tumor size reached 300 mm^3^, mice were given GNR preparation (1.5 mg/kg) either intratumorally or intravenousely in PBS solution every three weeks. Animals were exposed weekly to laser beam (50 W/cm^2^ for 2 min). Tumor tissue temperature was scanned using surface thermometer probe (Ugo Basile, Comerio, Italy); and temperature distribution was plotted against the radius of tumor tissue. Tumor size and overall survival were monitored for total of 7 weeks. Tumor size was determined using the formula: Volume = (Width^2^ x length)/2

Any animal showed massive weight loss, ascitis, change in motility or any other sign of morbidity was immediately sacrificed.

### Pathological examination

Histological examination for tumor tissues were performed according the lab routine protocol. Briefly, paraformaldhyde fixed tissues were embedded in paraffin wax. Cross vertical sections (5 µm) were obtained and after dewaxing and rehydration sections were stained with H&E.

### Statistical analysis

Data are presented as mean ± SEM. Analysis of variance (ANOVA) with LSD post hoc test was used for testing the significance using SPSS^®^ for windows, version 17.0.0. p<0.05 was taken as a cut off value for significance.

## Supporting Information

Figure S1 **Antitumor activity of GNRs coupled with laser induced photo plasmonic thermal therapy in EACC solid tumor bearing mice.** EACC tumor bearing mice were given gold NRs (1.5 mg/kg every three weeks) by I.V. (C) and I.T. (B) administration compared to PBS treated animals (A). Animals were exposed to laser plasmonic beam (50 W/cm^2^ for 2 min) every week. Thermal effect was measured immediately after the last laser exposure session and plotted (D).
(TIF)
