# Supplementary figures and images for: Tissue Distribution and Efficacy of Gold Nanorods Coupled with Laser Induced Photoplasmonic Therapy in Ehrlich Carcinoma Solid Tumor Model
Source: PLoS One. 2013 Oct 2;8(10):e76207. doi: 10.1371/journal.pone.0076207 (PMC3788801; doi:10.1371/journal.pone.0076207)

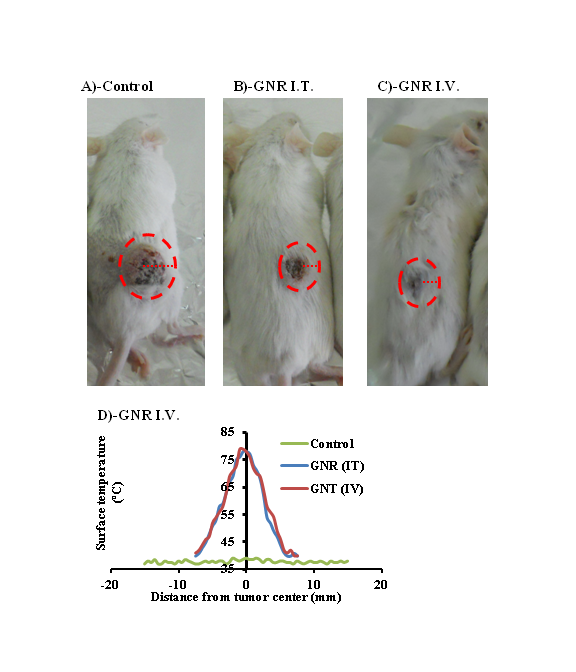

Supplement: Figure S1 — Antitumor activity of GNRs coupled with laser induced photo plasmonic thermal therapy in EACC solid tumor bearing mice. EACC tumor bearing mice were given gold NRs (1.5 mg/kg every three weeks) by I.V. (C) and I.T. (B) administration compared to PBS treated animals (A). Animals were exposed to laser plasmonic beam (50 W/cm2 for 2 min) every week. Thermal effect was measured immediately after the last laser exposure session and plotted (D). (TIF) [file pone.0076207.s001.tif]
